# Visit patterns at primary care centres and individual blood pressure level – a cross-sectional study

**DOI:** 10.1080/02813432.2019.1569369

**Published:** 2019-03-01

**Authors:** H. Ödesjö, S. Adamsson Eryd, S. Franzén, P. Hjerpe, K. Manhem, A. Rosengren, J. Thorn, S. Björck

**Affiliations:** a Department of Public Health and Community Medicine, Institute of Medicine, Sahlgrenska Academy, University of Gothenburg, Gothenburg, SE405 30, Sweden;; b Primary Health Care, Region Västra Götaland, Närhälsan Torslanda Vårdcentral, Torslanda, SE-423 34, Sweden;; c Department of Molecular and Clinical Medicine, Institute of Medicine, Sahlgrenska Academy, University of Gothenburg and Sahlgrenska University Hospital, Östra Hospital, Gothenburg, SE 416 50, Sweden;; d Centre of Registers Västra Götaland, Gothenburg, SE 413 45, Sweden;; e R&D Centre Skaraborg Primary Care, Skövde, Skövde, SE 541 30, Sweden

**Keywords:** Blood pressure, hypertension, nurse, primary health care, Sweden

## Abstract

**Objective:** Hypertension is a major cause of cardiovascular disease. Nevertheless, blood pressure (BP) is often inadequately treated. We studied visit patterns at primary health care centres (PHCCs) and their relation to individual BP control.

**Design and setting:** Cross-sectional register-based study on all patients with hypertension who visited 188 PHCCs in a Swedish region.

**Patients:** A total of 88,945 patients with uncomplicated hypertension age 40–79.

**Main outcome measures:** Odds ratio (OR) for the individual patient to achieve the BP target of ≤140/90 mmHg.

**Results:** Overall, 63% of patients had BP ≤ 140/90 mmHg (48% BP < 140/90). The PHCC that the patient was enrolled at and, as part of that, more nurse visits at PHCC level was associated with BP control, adjusted OR 1,10 (95% CI 1.01 to 1.21). Patients visiting PHCCs with the highest proportion of visits with nurses had an even higher chance of achieving the BP target, OR 1.19 (95% CI 1.07 to 1.32).

**Conclusions:** In a Swedish population of patients with hypertension, about half do not achieve recommended treatment goals. Organisation of PHCC and team care are known as factors influencing BP control. Our results suggests that a larger focus on PHCC organisation including nurse based care could improve hypertension care.

## Introduction

Hypertension is a major cause of cardiovascular disease and death. The prevalence of hypertension is high and BP control is unsatisfactory [1]. Less than half of Swedish patients achieve recommended BP target [2].

An ageing population and lower BP criteria have resulted in more patients being diagnosed with hypertension. The challenge for the health care system is immense, and effective ways of handling this large patient population are needed.

Besides individual pharmacological treatment, the effects of organisational measures on hypertension care have been studied. Team changes have proven efficient [3]. Assigning hypertension care to a nurse or other non-physician professional has been claimed to be effective [4]. A meta-analysis found lower systolic BP with nurse-led interventions combined with structured treatment algorithms [5]. Advanced-practice nurses in the Netherlands achieved equal or better results than other professionals [6]. In several countries task shifting from physician to nurse is applied to a larger extent [7]. Recent European guidelines on prevention of cardiovascular disease recommend teamwork [8].

Thus, previous studies suggest that structure of care is important in achieving BP control and that one component may be assigning part of the responsibility to someone other than the physician.

The aim of this study was to describe visit patterns as a measure of how care is structured at the PHCC level based on real-life data, as well as examine whether nurse-based care was associated with better BP control in patients with hypertension without complications.

## Materials and methods

We conducted a cross-sectional regional register study of patients with hypertension without major comorbidities in primary care.

### Study basis

Västra Götaland in south-west Sweden is a mixed urban and rural region with 1.6 million inhabitants (2015, 17% of the Swedish population).

The region has about 200 PHCCs after a national health care reform in October 2009. All 200 PHCCs, whether publicly or privately operated, are funded by tax revenue. All patients in the region are enrolled at a specific PHCC. Diabetes and asthma/COPD nurses are mandatory, but PHCCs are free to structure their hypertension care [9].

PHCCs report individual data for patients with hypertension to a regional primary care quality register.

National guidelines recommend a BP target of <140/90 mmHg, which complies with European guidelines [8].

### Patients

Patients with hypertension in the regional primary care quality register on 31 December 2015 for whom systolic BP data were available were eligible for inclusion in the study (*n* = 237,379).

**Figure 1.**
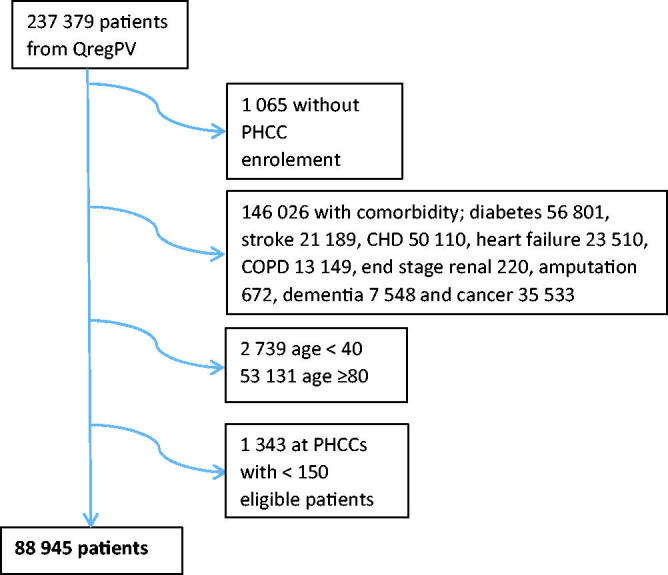
Flow diagram of patient inclusion and exclusions.

To reduce the influence of other diseases on visit patterns, we excluded patients <40 or ≥80 years old and those with concomitant disease, Figure 1. We also excluded patients without PHCC enrolment and those at 18 PHCCs with fewer than 150 eligible individuals. The average number of included patients per PHCC was 473 (156-1,200).

### Databases

This study was based on information obtained from linking a regional primary care quality register (QregPV), a regional administrative health care database (VEGA), the National Patient Register (NPR), the Prescribed Drug Register, and the Longitudinal Integration Database for Health Insurance and Labour Market Studies (LISA).

- QregPV contains information about all patients with a diagnosis of hypertension, diabetes, ischaemic heart disease, asthma or COPD in primary care in Västra Götaland. The register, which was launched in 2007 as a tool for quality assurance, includes data about systolic and diastolic BP, lipid levels, smoking, height, weight and waist circumference.
- VEGA covers all PHCC visits in Västra Götaland since 2001. Data include diagnoses, dates of visits, staff categories and PHCC enrolment.
- NPR covers diagnoses at the time of all hospital discharges since 1987, as well as outpatient visits in specialised care since 2001. This study included NPR data since 1997, when ICD-10 was adopted.
- The Prescribed Drug Register contains information about all prescriptions picked up since 1 July 2005.
- LISA, which is administered by Statistics Sweden, covers country of birth, marital status and education.

### Definitions and clinical examinations

The index date for each individual refers to the last BP entry before 31 December 2015. The variables used from QregPV are the last entries up to 900 days before the index date.

We collected information from the Prescribed Drug Register about antihypertensive and cardiovascular prescriptions picked up within 120 days before the index date: diuretics C03A-C03E, C09BA, C09DA, calcium channel blockers C07FB02, C08, C09BB, C09DB, beta adrenergic receptor blockers C07, renin-angiotensin system inhibitors C09, other antihypertensives C02, statins C10AA and acetylic salicylic acid B01AC06.

The variables from LISA were chosen in order of priority as of the index year, one year after, one year before, otherwise missing.

Excluded concomitant diseases with ICD-10 codes: Diabetes E10-14, Stroke I61, I63-64, Coronary heart disease I20-25, Heart failure I50, COPD J44, End stage renal disease Z94.0-Z49.2 and Z99.2, Amputation NFQ19, NFQ99, NGQ09, NGQ19, NGQ99, NHQ09, NHQ11-14, NHQ16-17 and NHQ99, Dementia G30.0-1, G30.8-9, F00-03, Cancer C0-9.

The regional recommendation is the mean value of two or three BP measurements after 5–10 minutes rest in the sitting or supine position with the arm at heart level. We used systolic BP ≥ 150 as a measure of poorly controlled hypertension.

### Assessment of variables

The PHCC explanatory variables used were as follows:

- PHCC mean number of visits to nurse: ${\mathrm{PHCC}}^{\mathrm{nurse}}$
- PHCC mean number of visits to physician: ${\mathrm{PHCC}}^{\mathrm{physician}}$
- Ratio of PHCC mean number of visits to nurse and physician: ${\mathrm{PHCC}}^{\mathrm{ratio}}=\frac{{\mathrm{PHCC}}^{\mathrm{nurse}}}{{\mathrm{PHCC}}^{\mathrm{physician}}}$

These variables are based on visits within 450 days before the index date with a hypertension diagnosis. To reduce the influence of other causes, patients with more than 12 visits to a nurse (*n* = 31) or physician (*n* = 8) were excluded when calculating PHCC explanatory variables.

BP was classified as measured by physician if only a physician visit on index date, otherwise by nurse. A BP limit of ≤140/90 mmHg was used as an outcome to avoid the effect of differences in rounding between physicians and nurses.

### Statistical methods

The aim of this study is explanatory rather than predictive and the methods chosen accordingly.

Descriptive statistics are presented as arithmetic mean and standard deviation for continuous variables and with frequencies and percentages for categorical variables.

A multi-level mixed model was used with the dichotomous dependent variable at the individual level of whether BP ≤ 140/90 mmHg. PHCC was modelled as a random factor. Fixed factors were age, sex, smoking, BMI, country of birth, marital status, educational level, visits, number of anti-hypertensive drugs and whether BP was measured by a physician or nurse.

Three different adjustment models were used: model 1 with adjustment for age and sex only, model 2 = model 1 plus BMI, smoking, country of birth, marital status, education and number of anti-hypertensive drugs, model 3 = model 2 plus number of individual visits with a physician or nurse and whether BP was measured by a physician or nurse.

OR for ${\mathrm{PHCC}}^{\mathrm{nurse}}$ and ${\mathrm{PHCC}}^{\mathrm{physician}}$ are presented as the increase/decrease in the likelihood that the patient would achieve the BP target for each extra mean visit to the PHCC. OR for ${\mathrm{PHCC}}^{\mathrm{ratio}}$ is presented as the increase/decrease in the likelihood that the patient would achieve the BP target if enrolled at a PHCC with more nurse than physician visits, ${\mathrm{PHCC}}^{\mathrm{ratio}}$>1 vs ≤1.

We also calculated median odds ratio (MOR) [10] with PHCC as a random factor in model 3 but without the primary explanatory PHCC variable, in order to interpret the magnitude of the PHCC variance/the PHCC effect on BP target achievement.

Inferential statistics are based on ten files, in which missing observations were imputed using multiple chained equations.

The statistical analyses used R 3.4.0 and SAS version 9.4 (SAS Institute, Cary, NC).

## Results

Descriptive data for included patients are shown in Table 1. The patients were broken down into two groups depending on PHCC visit characteristics. The mean BP at PHCCs with more nurse than physician visits was 1 mmHg lower. Furthermore, the percentage of patients with BP ≤ 140/90 mmHg was higher and the percentage with SBP ≥ 150 mmHg lower.

**Table 1.** Patient characteristics for all patients and broken down into groups depending on whether the patient is enrolled at a PHCC with more visits to physician than nurse (physician based) or the contrary (nurse based).

	Variable	All (*n* = 88945)	Physician based (*n* = 62990)	Nurse based (*n* = 25955)
Age		63.2 (9.4)	63.1 (9.5)	63.4 (9.3)
Sex	Female	48265 (54.3%)	34178 (54.3%)	14087 (54.3%)
SBP(mmHg)		137.5 (15.0)	137.8 (15.3)	136.9 (14.4)
DBP(mmHg)		82.6 (10.4)	83.0 (10.4)	81.7 (10.4)
BP <140/90 mmHg		42570 (47.9%)	29465 (46.8%)	13105 (50.5%)
BP < = 140/90 mmHg		56008 (63.0%)	39242 (62.3%)	16766 (64.6%)
SBP > = 150 mmHg		17238 (19.4%)	12663 (20.1%)	4575 (17.6%)
Smoking		10594 (13.8%)	7576 (13.9%)	3018 (13.7%)
BMI(kg/m2)		28.4 (5.0)	28.3 (5.0)	28.5 (5.0)
Total Cholesterol (mmol/L)		5.5 (1.1)	5.5 (1.1)	5.6 (1.1)
LDL Cholesterol (mmol/L)		3.5 (0.9)	3.5 (0.9)	3.5 (0.9)
Anti-hypertensive treatment		76413 (85.9%)	53619 (85.1%)	22794 (87.8%)
No. of anti-hypertensive drugs		1.5 (1.0)	1.5 (1.0)	1.5 (1.0)
Diuretics		26782 (30.1%)	18769 (29.8%)	8013 (30.9%)
Calcium channel blockers		30121 (33.9%)	21354 (33.9%)	8767 (33.8%)
Beta adrenergic receptor block		24208 (27.2%)	17181 (27.3%)	7027 (27.1%)
Renin-angiotensin system inh.		51459 (57.9%)	36196 (57.5%)	15263 (58.8%)
Born in non-Nordic country		8921 (10.0%)	7189 (11.4%)	1732 (6.7%)
Married/registered partner		37156 (41.8%)	26811 (42.6%)	10345 (39.9%)
Education level	Elementary school	23154 (26.2%)	15775 (25.2%)	7379 (28.6%)
Education level	High school	41229 (46.7%)	28894 (46.2%)	12335 (47.9%)
Education level	University	23895 (27.1%)	17843 (28.5%)	6052 (23.5%)
No. of visits to physician		1.3 (1.2)	1.5 (1.2)	0.9 (1.0)
No. of visits to nurse		0.8 (1.4)	0.6 (1.2)	1.5 (1.7)
Index date BP by physician		33578 (37.8%)	27492 (43.6%)	6086 (23.4%)

Notes: Mean (SD) and frequencies (%). BP: blood pressure; SBP: Systolic BP; DBP: Diastolic BP; BMI: body mass index; LDL: low density lipoprotein.

If visiting a nurse-based PHCC, the chance of having picked up an antihypertensive prescription was higher (1.21 (1.16–1.27)), as well as being treated with diuretics (1.05 (1.01–1.08)) or renin-angiotensin system inhibitors (1.06 (1.03–1.09)), adjusted for age and sex.

**Figure 2.**
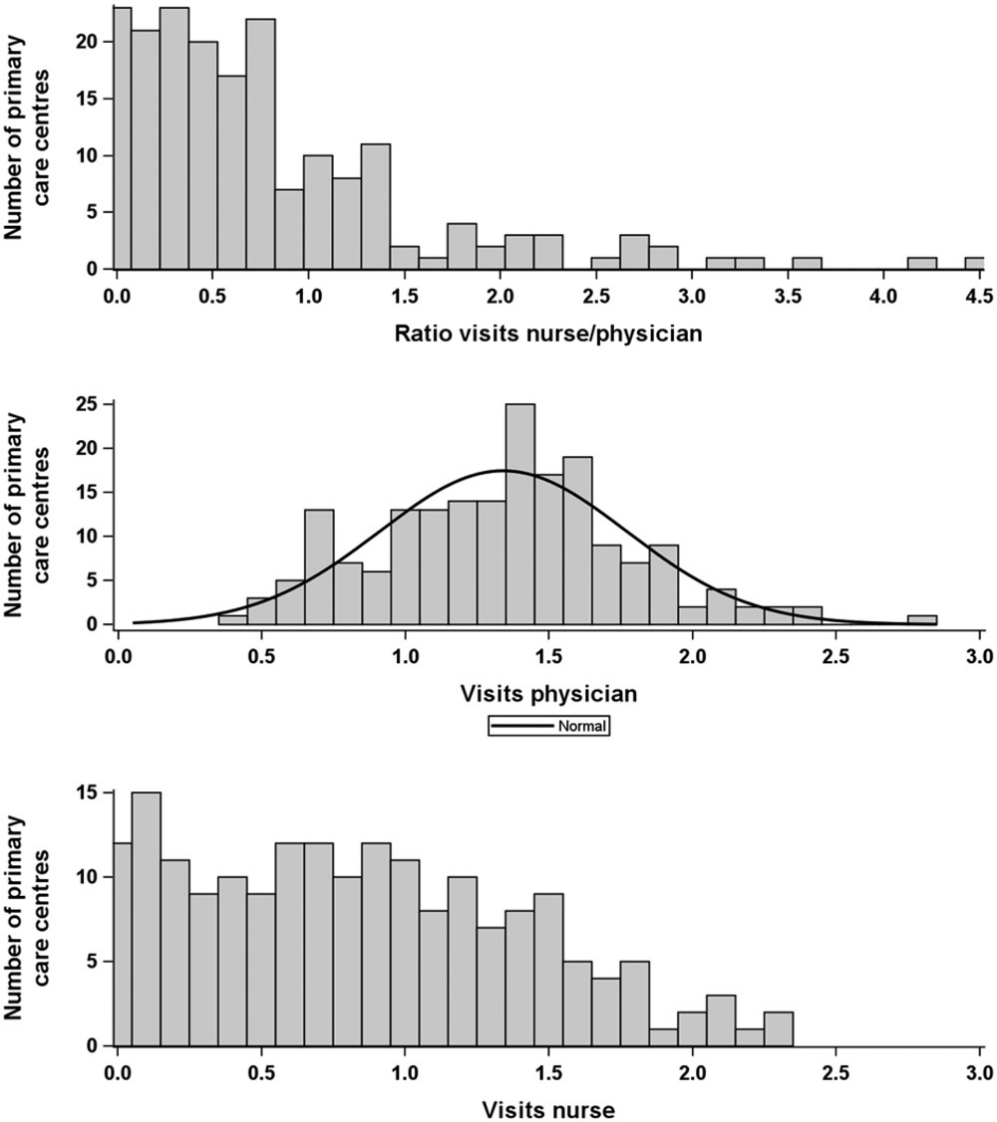
Distributions of ratio between number of visits to nurse and physician (top) and mean number of visits to physician (middle) or nurse (bottom) for the 188 included PHCCs.

Only 52 of 188 PHCCs (30% of patients) were nurse-based. At 118 PHCCs, the mean number of visits with a nurse was less than 1; see the PHCC distribution of visits in Figure 2. Mean ratio visits nurse/physician in nurse-based PHCCs was 1.84 ± 0.83 vs 0.41 ± 0.28 in physician-based PHCCs (mean ± SD).

**Figure 3.**
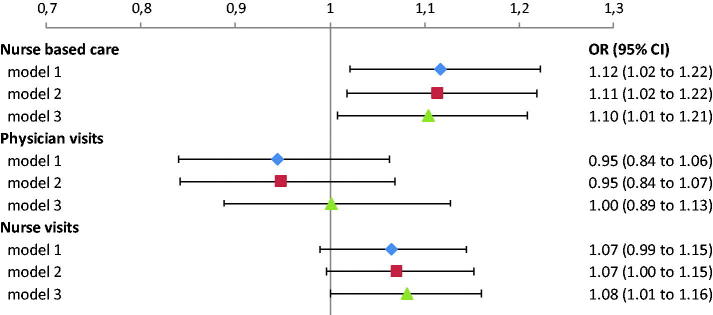
OR (odds ratio) that the patient would achieve BP ≤140/90 mmHg: at nurse-based PHCCs (the number of visits to nurse is larger than the number of visits to physician) and with an increased mean number of visits to physician or nurse at the PHCC (the OR represents an increase of one in the mean number of visits to physician and nurse respectively). Three different adjustment models were used: model 1 with adjustment for age and sex only, model 2 = model 1 plus BMI, smoking, country of birth, marital status, education and number of anti-hypertensive drugs, model 3 = model 2 plus number of individual visits with a physician or nurse and whether BP was measured by a physician or nurse.

Patients at PHCCs with nurse-based care had a 10% higher chance of achieving the BP target (OR 1.10 (1.01–1.21)), Figure 3. We could not show that the risk of having poorly controlled hypertension (SBP ≥150 mmHg) was lower at a PHCCs with more visits with nurses 0.97 (0.86–1.09). The results above were stable to adjustments in model assumptions. Without exclusion for major comorbidity, the OR for achieving the BP target for nurse-based care was 1.13 (1.01–1.25). When using the BP target <140/90 mmHg, instead of < =140/90, OR was 1.13 (1.03–1.24). Although patients with cardiovascular diagnoses were excluded, 21,738 patients were taking medications for such diseases. Adding these medications as covariates to model 3 did not change the results.

The median increased odds of reaching BP target if a patient moved from a low- to a high-performing PHCC was 1.45 whereof, according to results above, approximately 22% (10/45) could be associated with nurse-based care.

**Figure 4.**
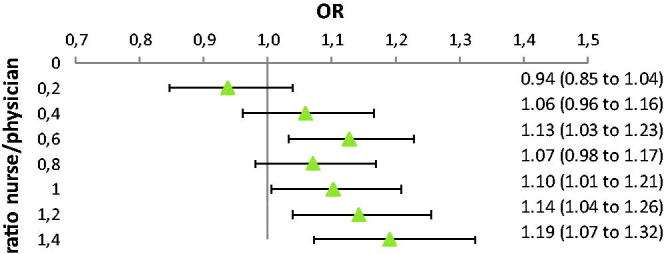
OR (odds ratio) that a patient would achieve BP ≤ 140/90 mmHg calculated using different ratios between nurse and physician visits at the 188 PHCCs. The OR represents the patients visiting PHCCs with a nurse/physician ratio higher than the number on the y-axes. A value of 1 on the y-axes is the same analysis as in nurse based care in Figure 3 above. The full adjustment model (model 3) was used.

Nurse-based care was associated with higher BP target achievement and the OR increased accordingly. Patients enrolled at PHCCs with the highest proportion of nurse visits had a 19% higher OR of achieving the BP target, Figure 4. A crude Pearson correlation analysis with PHCC proportion of patients reaching BP target vs ${\mathrm{PHCC}}^{\mathrm{ratio}}$ showed a correlation of 0.22 (*p* = 0.003)

## Discussion

PHCC enrolment and, as part of that, nurse based care was associated with better BP control. We found a large variation in visit patterns for patients with uncomplicated hypertension among PHCCs in terms of both frequency and the ratio between nurses and physicians. PHCC enrolment was associated with BP target achievement and it is plausible that part of this association was explained by examined visit patterns. The higher the percentage of nurse visits at the PHCC, the greater the likelihood that a patient would achieve the BP target.

One strength of our study is that all patients with hypertension in primary care are included in a Swedish region. Another strength is extensive data about visits, comorbidity and picked up medications.

One limitation of the study is that we had no information regarding the structure of hypertension care from each PHCC but used visit patterns instead. Another limitation involved the way that BP was measured, but information was included about the professional who presumably measured BP on the index date. BP may also have been measured not as part of hypertension control but to assess other complaints, etc. To minimise this bias, we used BP entries only when the visit had a diagnosis of hypertension. The last BP measurement has been shown to correlate well with the mean of three entries over the period of a year [11].

We chose to focus on patients without cardiovascular disease, diabetes or other serious conditions. One reason is that this large and growing patient population is easily overlooked. Another reason is that patients with chronic conditions may have more physician visits, which would compromise the interpretation of the nurse/physician ratio.

Barriers to hypertension control can be related to both the patient and the health care professional [12]. Examples of patient barriers are lack of knowledge, stress, depression and anxiety as reasons for postponing adoption of a healthier lifestyle. Supporting patients´ self-management in hypertension care has been studied and could be efficient [13,14]. In a survey study among primary care the most prominent physician related barrier was found to be the acceptance of a BP level higher than the recommended target [15]. Among physician-related reasons are also competing medical issues. Our results that showed better BP control with nurse-based hypertension care are probably related to factors described in these studies.

Various quality improvement strategies for hypertension care have been identified that target the patient, professional (team change) or healthcare system [3]. A systematic review found that team changes are effective where hypertension care was assigned to a nurse [4]. Meta-analyses and review articles have shown that nurse-led interventions and structured treatment algorithms can improve BP control [5,16,17]. In several countries but to a lesser extent in Sweden, task shifting from physician to nurse is common [7]. The task shifting can include for example prescriptions, treatment and referrals. Our study supports the hypothesis that team-based care is superior to standard approaches. In our study, nurse-based care includes a team since anti-hypertensive drugs in Sweden can be prescribed only by a physician.

We cannot fully explain better BP target achievement with nurse-based care. Patients at nurse-based PHCCs had slightly more visits altogether and the same mean number of medications but picked up more renin-angiotensin system inhibitors and diuretics. The chance of having picked up antihypertensive prescriptions was higher at nurse-based PHCCs. There may also be differences in dosage and differences related to more intensive lifestyle interventions, etc., as shown previously [18]. Another explanation may be that more structured treatment algorithms, which have been shown to be successful in several studies, were used. We believe that shifting a task from a physician to a nurse is plausibly accompanied by more strict treatment algorithms. Internationally task shifting is more common than in Sweden and a more extensive task shifting could be a potential way of taking care of this large patient group.

## Conclusions

In conclusion, fewer than 50% of patients with hypertension had BP below 140/90 mmHg. In our study, PHCC enrolment and as part of that nurse-based care was associated with better BP control. Although previous studies have shown that team-based care is effective, and it is recommended in European guidelines, we found that more than 2/3 of PHCCs based their hypertension care mainly on physician visits. Thus, it is important to consider structural factors when planning hypertension care.
